# Prostate-specific antigen (PSA) and distress: - a cross-sectional nationwide survey in men with prostate cancer in Sweden

**DOI:** 10.1186/s12894-019-0493-1

**Published:** 2019-07-12

**Authors:** Ulrika Rönningås, Per Fransson, Maja Holm, Agneta Wennman-Larsen

**Affiliations:** 1 Division of Insurance Medicine, Department of Clinical Neuroscience, Karolinska Institutet, 171 77 Stockholm, Sweden; 2 Department of Oncology, Sundsvall County Hospital, 851 86 Sundsvall, Sweden; 3 Department of Nursing Science, Sophiahemmet University, 114 86 Stockholm, Sweden; 4 Department of Nursing, Umeå University, 901 87 Umeå, Sweden; 5 Cancercentrum, Norrlands University Hospital, 901 85 Umeå, Sweden

**Keywords:** Prostate cancer, Prostatic neoplasm, PSA, Prostate-specific antigen, Distress, Quality of life

## Abstract

**Background:**

The prostate-specific antigen (PSA) -value is often used during the prostate cancer trajectory as a marker of progression or response to treatment. Concerns about PSA-values are often expressed by patients in clinical situations. Today there is a lack of larger studies that have investigated the association between PSA-value and distress. The aim was to investigate the association between PSA-values and distress adjusted for sociodemographic factors, hormonal therapy and quality of life (QoL), among men with prostate cancer.

**Methods:**

In this cross-sectional survey of 3165 men with prostate cancer, members of the Swedish Prostate Cancer Federation, answered questions about sociodemographic factors, PSA, distress, QoL and treatments. Descriptive statistics, and bivariate and multivariable analyses were performed. The result was presented based on four PSA-value groups: 0–19, 20–99, 100–999, and ≥ 1000 ng/ml.

**Results:**

Of the men, 53% experienced distress. An association between distress and PSA-values was found where higher PSA-values were associated with higher OR:s for experiencing distress in the different PSA-groups: 0–19 ng/ml (ref 1), 20–99 ng/ml (OR 1.25, 95% CI 1.01–1.55), 100–999 ng/ml (OR 1.47, 95% CI 1.12–1.94), ≥1000 ng/ml (OR 1.77, 95% CI 1.11–2.85). These associations were adjusted for sociodemographic factors and hormonal therapy. In the multivariable analyses, beside PSA-values, higher levels of distress were associated with being without partner or hormonal therapy. When adding QoL in the multivariable analysis, the association between PSA and distress did not remain significant.

**Conclusion:**

These results indicate that the PSA-values are associated with distress, especially for those with higher values. However, to be able to support these men, continued research is needed to gain more knowledge about the mechanisms behind the association between emotional distress and PSA-values.

## Background

Among Swedish men, prostate cancer is the most common type of cancer comprising 33% of all cancer cases [1]. The prostate-specific antigen (PSA)-test is not specific to prostate cancer, but the value may increase when cancer is present and the PSA-test is used for detection of cancer. The value is used in diagnostics to evaluate the severity of the tumor, but is also used throughout the disease trajectory during monitoring and treatment [2].

The PSA-values are often divided into different PSA-groups and the groupings are decisive in risk analysis at detection, recurrence and progression of the prostate cancer. Despite the relevance of measuring PSA in all stages of the disease few studies have been conducted regarding the importance and impact that the PSA-values have for the men with prostate cancer. The existing studies are small and mainly qualitative and most report these findings as incidental. Interviews with men with prostate cancer receiving curative treatment showed that PSA-tests and values dominated the narratives of many participants’. The men tended to focus on the numeric value of the PSA and they often compared PSA-values with each other [3]. Roth and Passik [4] introduces the concept of a specific anxiety related to PSA, *PSA-anxiety*, among men with prostate cancer when studying overall anxiety in this group. Another study [5] showed that survivors of prostate cancer with high scores on fear of recurrence also had higher levels of PSA-anxiety and lower health-related quality of life (QoL).

In one interview study, men with localized prostate cancer described the PSA-tests as a lifeline and that they had a feeling of being healthy when the PSA-value was low and stable [6]. Shen et al. [7] interviewed men with biochemical recurrence in relation to their experience and affective responses regarding increasing PSA-values and treatment options. Most men experienced more worry and fear of death when they learned about the rising PSA-values than they did when their cancer was initially diagnosed. They were also more worried before a physician’s appointment if they knew PSA-values were to be discussed. Many of them kept track of the PSA-values, checked the doubling time and had determined their own PSA-limits when they thought a new treatment was to begin.

In a small questionnaire study [8] on how patients with prostate cancer knew if their disease was progressing, 77% of the 52 men reported PSA-levels as one of the indicators. The study showed that many men with prostate cancer defined changes in disease through their PSA-value instead of their physical and functional status. For some men a decrease in the PSA-value was a more important goal than a reduction of disease symptoms [8]. In an interview study of men with castration-resistant prostate cancer the researchers found that the men graded PSA-anxiety and fatigue as being most important when grading complications and QoL issues [9]. Nanton, Dochertry, Meystre and Dale [10] suggest that there is an uncertainty about what happens during the prostate cancer journey regarding the disease and treatment, which may generate anxiety and distress. Worry and anxiety as signs of distress may be related to fear of the future [10]. Along the cancer continuum, distress is frequent, and several factors, have been shown to be involved in terms of distress in cancer, such as psychological, social and spiritual factors [11]. Symptoms of distress, e.g., worry and fear about the future, thoughts of illness and death, and also concerns about physical symptoms, can be disabling and lead to depression, anxiety and panic disorders [11]. Other factors that have been shown to be associated with distress in men with prostate cancer are ongoing hormone treatment [12], and younger age, where younger cancer survivors tend to have higher fear of recurrence than older cancer survivors [13]. Another study suggests a more complex situation, where overall distress and anxiety decreases with age at the same time as depressive symptoms increase [14].

In conclusion, there are few studies about PSA-values from the menʼs perspective and most of them are either small quantitative studies or interview studies. In addition, the PSA-value and its impact on the men have mostly been reported as incidental findings. As mentioned, some studies have shown that men with prostate cancer may experience PSA-anxiety in relation to PSA-tests, when having increasing PSA-values and before attending appointments with their physician [3, 5, 7–9]. However, to our knowledge, there is a lack of larger studies that have investigated the association between PSA-values and distress, and what importance the different levels of PSA have, regarding the men’s distress, especially when the PSA-values are high or extremely high.

Therefore, the aim of this study was to investigate the association between PSA-value and distress among men with prostate cancer, adjusted for sociodemographic factors, hormonal therapy (surgical or medical) and overall QoL.

## Methods

Data were obtained from an overall cross-sectional study where a questionnaire was sent to members of the Swedish Prostate Cancer Federation (SPCF) in collaboration with one of the authors (PF) in 2012 on the initiative of the SPCF [15]. The questionnaire was distributed to all 6389 members. Members in the federation consist of men with prostate cancer and their family members/relatives, professionals as well as individuals who join the federation in support of it. Out of the 6389 distributed questionnaires, 3512 (55%) were returned. Of the returned questionnaires 3165 (90%) were received from members who reported that they had been diagnosed with prostate cancer. Hence, answers from these 3165 men were used in the analysis reported here.

## Data collection

Some questions for this study was taken from a validated questionnaire developed by Fransson, Tavelin and Widmark [16] whereas others, were developed for the project in close cooperation with the SPCF [15]. The questions used in the present study are concerning age, marital status, level of education, time since diagnosis, type of treatments, the latest PSA-value, distress and overall QoL. Distress was measured by the question “Do you have any problems with worry/anxiety/feelings of depression?” with the response options “Never”, “Sometimes”, “Often” and “Always”. QoL was measured by the question “How would you estimate your QoL today?” with the answers on a numeric rating-scale from 0, “Very bad” to 10, “Excellent”.

For the multivariable analysis, distress was dichotomized into: those who never experienced distress and those who experienced distress sometimes/often/always. QoL was dichotomized by the median (7) into: 0–6 and 7–10. Age was dichotomized by the median (73) into: 41–73 years and 74–95 years. Marital status was dichotomized into: married/cohabiting/in a relationship and divorced/widowed/single. Education level was categorized in three groups: elementary school, high school, and university. Years since prostate cancer diagnosis were dichotomized by the median (6) into: 0–6, and 7–43 years. Hormonal therapy was dichotomized into: “no hormonal therapy (no surgical or ongoing medical)” and “hormonal therapy (surgical or ongoing medical)”. PSA-values were categorized into four groups according to a grouping suggested by Koo et al. [17]: 0–19 ng/ml; 20–99 ng/ml; 100–999 ng/ml; and ≥ 1000 ng/ml, −where the values ≥1000 ng/ml are considered to be extremely high.

### Data analysis

Data analysis was conducted using IBM SPSS 23 (Statistical Package for the Social Science). Data were analysed using descriptive statistics regarding age, marital status, education, years since diagnosis, hormonal therapy and latest known PSA-value. Listwise deletion was applied to manage missing values. A logistic regression analysis with 95% CI was used to explore the association between distress and latest known PSA-value adjusted for sociodemographic factors, hormonal therapy and overall QoL. The logistic regression analysis was performed in three models; first as a bivariate analysis (Model 1). In Model 2, all significant variables (*p <* 0.05) from Model 1 were included simultaneously except for QoL. This strategy was chosen because distress is assumed to be part of the concept of overall QoL to avoid over-adjustment in the first step. In Model 3, QoL was included for the purpose of adjusting for wellbeing other than distress, including physical aspects of wellbeing. Model 3 should be interpreted carefully because there is a theoretical overlap between the concepts of distress and QoL, where distress may be a part of the overall concept of QoL [18].

## Results

### Sample characteristics

Of the 3165 men, the median age was 73 years (min–max, 41–95), and 80% (2609/3165) of the participants were in a relationship. Regarding the participantsʼ levels of education, 38% (1213/3165) had completed elementary school, 28% (886/3165) had completed high school and 30% (937/3165) had a university education (Table 1).

**Table 1:** Sociodemographic and medical characteristics of 3165 men with prostate cancer: frequencies, percentages, mean, standard deviation (SD), median, range

Age (years)	Mean (*SD*)	72.8 (*7.2*)	
	Median (*Interquartile*)	73 (*10*)	
	Missing	*n* = 24	0.8%
	Range	41–95	
Years since diagnosis (years)	Mean (*SD*)	6.9 (*4.4*)	
	Median (*Interquartile*)	6 (*5*)	
	Missing	*n* = 114	3.6%
	Range	0–43	
Quality of Life^a^	Mean	6.9	
	Median	7	
	Quality of Life 0–6	*n* = 1112	35.7%
	Quality of Life 7–10	*n* = 2006	64.3%
	Missing	*n* = 47	1.5%
		*n*	%^b^
Marital status	Married/cohabiting	2525	8.0
	Divorced	78	2.6
	Widowed	182	6.1
	Single	101	3.4
	In a relationship	84	2.8
	Missing	195	6.2
Education	Elementary school	1213	40.0
	High school	886	29.2
	University	937	30.9
	Missing	129	4.1
PSA – latest value (ng/ml)	0–19	2336	73.8
	20–99	473	14.9
	100–999	267	8.4
	1000–	89	2.8
	Missing	0	0.0
Hormonal therapy	No	2556	81.8
	Yes	609	19.2
	Missing	0	0.0
Distress	Never	1455	46.6
	Sometimes	1468	47.0
	Often	166	5.3
	Always	31	1.0
	Missing	45	1.4

^a^Higher values indicate better QoL

^b^Totals i some cases do not amount to 100% due to the use of rounding

The time since diagnosis of prostate cancer varied between 0 and 43 years, with a mean value of almost 7 years and a median of 6 years. All the men had reported their latest PSA-value. The largest group (74%) had relatively low PSA-values (0–19 ng/ml), while 3% had extremely high PSA-values (≥1000 ng/ml). About 46% (1455/3165) stated that they “never” experienced distress, while about 53% (1665/3165) experienced distress “sometimes”, “often” or “always”. Most men (64%, 2006/3165), reported good QoL (Table 1).

### Associations between PSA-level and distress

In the bivariate analysis (Model 1), higher PSA-values were significantly associated with more distress compared to lower levels of PSA (0–19 ng/ml) (Table 2). Not being in a relationship, having ongoing hormonal therapy or low overall QoL were also associated with higher levels of distress, while those with a university-level education had less distress compared to those with elementary school (Table 2). No association was found between distress and the number of years since the prostate cancer diagnosis, or age (Table 2).

**Table 2:** Crude and adjusted odds ratios (OR) with 95% confidence interval (95%CI), for associations between distress and PSA, sociodemographic factors, hormonal therapy (surgical or medical) and overall QoL (*n* = 3165)

Variable	Model 1^a^			Model 2^b^			Model 3^c^		
	OR	(95% CI)	*p*	OR	(95% CI)	*p*	OR	(95% CI)	*p*
Age *n* = 3141									
41–73	1			-			-		
74–95	0.90	(0.78–1.04)	0.14						
Years since diagnosis *n* = 3051									
0–6	1			-			-		
7–43	1.10	(0.96–1.27)	0.18						
Quality of life *n* = 3118									
7–10	1			-			1		
0–6	4.49	(3.81–5.29)	**0.00**				4.33	(3.62–5.17)	**0.00**
Marital status *n* = 2970									
Married/cohabiting/in a relationship	1			1			1		
Single/widowed/divorced	1.50	(1.19–1.88)	**0.00**	1.40	(1.10–1.77)	**0.01**	1.16	(0.90–1.49)	0.25
Education *n* = 3036									
Elementary school	1			1			1		
High school	1.04	(0.87–1.24)	0.83	1.06	(0.89–1.28)	0.50	1.21	(1.00–1.47)	**0.05**
University	0.83	(0.70–0.99)	**0.02**	0.85	(0.71–1.01)	0.07	1.00	(0.83–1.21)	0.98
PSA (ng/ml) *n* = 3165									
0–19	1			1			1		
20–99	1.36	(1.11–1.66)	**0.00**	1.25	(1.01–1.55)	**0.04**	1.21	(0.96–1.51)	0.10
100–999	1.54	(1.19–2.01)	**0.00**	1.47	(1.12–1.94)	**0.01**	1.25	(0.93–1.68)	0.14
1000–	2.19	(1.38–3.47)	**0.00**	1.77	(1.11–2.85)	**0.02**	1.17	(0.70–1.94)	0.55
Hormonal therapy *n* = 3165									
No	1			1			1		
Yes	1.42	(1.18–1.71)	**0.00**	1.35	(1.11–1.65)	**0.00**	1.12	(0.91–1.39)	0.28

*p* ≤ 0.05, significant values in bold

^a^Model 1: Bivariate association between all variables and distress

^b^Model 2: Association between PSA-value and distress adjusted for all significant independent variables in Model 1 excluding QoL

^c^Model 3: Association between PSA-values and distress adjusted for all significant independent variables in Model 1 also including QoL

In the first step in the multivariable analysis (Model 2) when adjusted for education, marital status and hormonal therapy but not QoL, the odds ratio (OR) for having distress significantly increased with higher PSA-values (PSA-group 20–99 ng/ml, OR 1.25 (95% CI 1.01–1.55), PSA-group 100–999 ng/ml, OR 1.47 (95% CI 1.12–1.94), PSA group ≥1000 ng/ml, OR 1.77 (95% CI 1.11–2.85)). The significant association between marital status, hormonal therapy and distress also remained (Table 2). In Model 3, in which QoL was included, there was an association between QoL and distress, OR 4.33 (95% CI 3.62–5.17) while the association between distress and PSA-values and the association between distress and marital status, ongoing hormonal therapy and PSA-values was not significant (Table 2).

## Discussion

The results show that higher PSA-values were associated with higher likelihood for experiencing distress. Not living in a relationship and hormonal therapy were factors also associated with reported distress as well as overall QoL.

About half of the men in this study experienced distress at least sometimes and there was an association between distress and PSA-values in the groups, PSA 20–99 ng/ml, PSA 100–999 ng/ml and PSA ≥ 1000 ng/ml. The association between PSA-value and distress was positive, i.e. higher PSA-values were associated with higher OR:s for experiencing distress. Lofters et al.ʼs [8] results showed that PSA-values were strongly correlated with anxiety. Medium to severe anxiety before receiving the results of a PSA-test was also reported by a third of the men with metastatic prostate cancer in their study [8]. An association between rising PSA-values and increasing anxiety among men 6–18 months after prostatectomy has also been shown [19]. PSA-values seem to affect men diagnosed with prostate cancer in a similar way in which the cancer antigen (CA) 125-values affect women diagnosed with gynecological cancer. Parker et al. [20] described that women often knew their CA 125-value, many wrote the value down and tracked the values over time and they also reported that the values affected their mood. Parker et al.ʼs study also showed that the more metastases the women had the more they were preoccupied with the CA 125-values [20]. In this study, we did not have data on metastases and can therefore only speculate on whether men with metastases, who probably have higher PSA-values, are more preoccupied with their PSA than men without metastases. However, in the group of men with extremely high PSA-values (≥1000), we do know that they have a metastatic prostate cancer, even if not detected, and the disease may affect the men, with more physical symptoms as well. Distress induced by higher PSA-values may be related to fear of recurrence and progression, and the laboratory referral for PSA-test may eventually work as a trigger for fear of recurrence or progression of the disease. Results from a study by Simard, Simard and Ivers (2013) showed that around 10% of patients diagnosed with cancer reported high scores for fear of recurrence and, and also reacted more to “triggers” for fear of recurrence as well as had more psychological distress [21]. “Triggers” in this case are things leading to intrusive thoughts and fear of recurrence, for example, reading a newspaper and seeing an advertisement about cancer treatment or during a physical examination [13, 21]. When patients with metastatic disease were asked about their opinion about discontinuing PSA-tests, more than 50% responded that it would feel as though the physician had given up on them; only one patient thought it would be a relief [8]. In the questionnaire for the present study, all men reported their last PSA-value, even those who reported a longer period since their diagnosis. Together with hormonal therapy, it was the only variable with no missing data, which may indicate the importance of PSA-values and the impact that the hormonal therapy had on these men.

In both the bivariate and in the adjusted analyses, it was shown that other factors besides PSA-values were also associated with distress, e.g., men who lived without a partner had a higher likelihood of being distressed. One explanation may be that; even if men who live in a relationship and who have high PSA-values may be distressed, they may also receive more support from their partner, which could in turn reduce the level of distress. In a previous interview study of men with prostate cancer who were living without a partner, a higher need for information and support regarding treatment alternatives was also found when compared to men who were living in a relationship [22]. However, the level of partner support may also affect the experienced distress, where men reporting low partner support have been shown to experience the same level of distress as those who do not have a close relationship [23]. Men not living in a relationship experienced more social isolation and many of them did not seek support actively. Nonetheless, the authors described that during interviews descriptions of anxiety and fear of dying appeared even though the men did not always recognise the emotional distress affecting them [24].

Hormonal therapy was also a factor associated with distress in this study. This result is in line with those of Sharpley, Bitsika, Wooten and Christie [25], which show that men receiving hormonal therapy had higher scores regarding anxiety and depression, compared to men who had completed the hormonal therapy or who had never had hormonal therapy [25]. It would have been interesting to know which type of hormonal therapy was given to the men in the present study and at what stage of the disease. Distress may be experienced differently if the hormonal therapy is adjuvant or if it has a palliative intent. Unfortunately, we did not have access to data on type of hormonal therapy for the men in the present study, thus this could be a question for future studies.

When adding QoL into the models the association between distress and PSA-levels were no longer significant. This may be due to a possible dependency between QoL and distress, where QoL is an overall concept that also covers aspects of distress. Overall QoL had a stronger association with distress than PSA-values, which may be interpreted as the notion that there are more aspects of health that may affect the level of distress in this group of patients. Dale et al. [18] describe that it is possible that an increasing PSA-value may increase the worry and anxiety-level, which in turn may affect health-related QoL [18]. When comparing early-stage prostate cancer and advanced-stage prostate cancer with bone metastases, those in the late-stage group had more anxiety [26]. The PSA-value is also often important in treatment evaluation and may also in that sense be related to overall QoL. Distress could also be another aspect affecting QoL. As mentioned earlier, distress may be a part of the overall concept of QoL because there is a theoretical overlap between the concepts of distress and QoL [18] and therefore the results of this analysis should be interpreted cautiously.

A bit surprisingly, the present study did not show an association between distress and age, whereas earlier studies have shown that in newly diagnosed men with localized prostate cancer, younger age was associated with greater distress [27] and younger cancer survivors also had a higher fear of cancer recurrence [13], which may also be associated with distress. Previous results based on the same sample as in the present study have also shown that younger men had more thoughts of suicide than older men with prostate cancer [15]. The rather high age (mean 72.8) with few young men (SD 7) in the present sample could however explain the lack of association between age and distress.

A clinical implication of the results in the present study is that there may be a need to pay more attention to men living without a partner and/or who receive hormonal therapy (surgical or ongoing medical) when awaiting PSA results. This may be an important aspect to consider for the treating physician, and for the patient access to a specialist nurse may also be helpful in order to receive relevant information and support [28].

### Strengths and limitations

One strength of this study is the large nationwide sample, providing a large spread in age, 41–95 years, and in years since diagnosis, 0–43 years. Hence, the data include various generations and were drawn both from newly diagnosed men and those diagnosed many years ago. Although, men who are members of the SPCF may be a selected group, earlier research [29] has shown that data from members of prostate cancer associations are likely to correspond with data from other studies within the wider prostate cancer population regarding symptoms, QoL and disease status [29]. Earlier research has also shown that men do not become members of patient associations due to distress or, as for women, due to psychosocial needs related to having cancer. For men with prostate cancer, the motives were rather that they wanted the information and activities the associations could provide [30]. Data from prostate cancer patients’ associations have also been used in previous studies of this population in the Nordic Countries [29, 31, 32]. A limitation in this study is however that we do not know how many of the non-responders were men with prostate cancer and how many that were supportive members of the federation and therefore we cannot estimate the actual response rate. There is a possibility that more supportive members were non-responders due to the cancer-related questions.

A limitation of this study is also that the questions in the questionnaire were not specifically developed for the aim of the present study. One example is that distress was measured by the question “Do you have any problems with worry/anxiety/feelings of depression?” which covers three aspects of distress. This may be problematic if only one of the aspects were experienced. A strength may, on the other hand, be that the question covers many aspects of overall distress, thus it does not exclude men who experienced any of the aspects.

It would also have been interesting to control for data on other factors that may cause distress, such as physical symptoms and/or comorbidity. It would have been interesting to compare other aspects of wellbeing than QoL, i.e. performance status or physical functioning.

It would also have been advantageous to verify data on disease status and PSA-values from medical records which was not possible due to the nationwide sample with anonymous answers. There may be a possibility that the distress would have been even more prominent when measured closer to the PSA-testing. Another strength would also have been to have data on stage or status of the disease. In this case, a low PSA-level may both indicate that the disease is cured, in a stable, controlled phase, e.g., after initial treatment, or in a state where successful life-prolonging treatment is given. Thus, by treatment and treatment outcome, patients may move between the different PSA-groups. However, we do know that those with the highest PSA-values do have a more severe disease.

## Conclusion

An association between PSA-values and distress was found, where higher PSA-values were associated with higher likelihood of distress. Other factors, such as hormonal therapy and not living in a relationship, were also found to be associated with distress, as was overall QoL. However, to be able to support these men to reduce distress related to PSA-values, which is also a marker of progression, more detailed knowledge is needed on the complex mechanisms behind the experienced distress, and also on the role of PSA-values for understanding the disease from the menʼs perspective.

## Data Availability

The data that support the findings from this study will not be available for data sharing due to the information given to the participants and the approval from the Ethics Committee.

## Abbreviations

PSA: Prostate Specific Antigen
QoL: Quality of Life
SPCF: Swedish Prostate Cancer Federation
